# High‐pressure acidified steaming with varied citric acid dosing can successfully detoxify mycotoxins

**DOI:** 10.1002/fsn3.3324

**Published:** 2023-03-17

**Authors:** Chinaza Godswill Awuchi, Onyenibe Sarah Nwozo, Patrick Maduabuchi Aja, Grace Akinyi Odongo

**Affiliations:** ^1^ Department of Biochemistry Kampala International University Bushenyi Uganda; ^2^ School of Natural and Applied Sciences Kampala International University Kampala Uganda; ^3^ Department of Biochemistry University of Ibadan Ibadan Nigeria; ^4^ Department of Biochemistry Ebonyi State University Abakaliki Nigeria; ^5^ International Agency for Research on Cancer World Health Organization Lyon France

**Keywords:** aflatoxins, citrinin, high‐pressure acidified steaming using citric acid, mycotoxin detoxification, mycotoxins, ochratoxins

## Abstract

Mycotoxins are toxic fungal metabolites that exert various toxicities, including leading to death in lethal doses. This study developed a novel high‐pressure acidified steaming (HPAS) for detoxification of mycotoxins in foods and feed. The raw materials, maize and peanut/groundnut, were used for the study. The samples were separated into raw and processed categories. Processed samples were treated using HPAS at different citric acid concentrations (CCC) adjusted to pH 4.0, 4.5, and 5.0. The enzyme‐linked immunosorbent assay (ELISA) kit method for mycotoxins analysis was used to determine the levels of mycotoxins in the grains, with specific focus on total aflatoxins (AT), aflatoxins B_1_ (AFB_1_), aflatoxin G_1_ (AFG_1_), ochratoxin A (OTA), and citrinin. The mean values of the AT, AFB_1_, AFG_1_, OTA, and citrinin in the raw samples were 10.06 ± 0.02, 8.21 ± 0.01, 6.79 ± 0.00, 8.11 ± 0.02, and 7.39 ± 0.01 μg/kg for maize, respectively (*p* ≤ .05); and for groundnut (peanut), they were 8.11 ± 0.01, 4.88 ± 0.01, 7.04 ± 0.02, 6.75 ± 0.01, and 4.71 ± 0.00 μg/kg, respectively. At CCC adjusted to pH 5.0, the AT, AFB_1_, AFG_1_, OTA, and citrinin in the samples significantly reduced by 30%–51% and 17%–38% for maize and groundnut, respectively, and were reduced to 28%–100% when CCC was adjusted to pH 4.5 and 4.0 (*p* ≤ .05). The HPAS process either completely detoxified the mycotoxins or at least reduced them to levels below the maximum limits of 4.00–6.00, 2.00, 2.00, 5.00, and 100 μg/kg for AT, AFB_1_, AFG_1_, OTA, and citrinin, respectively, set by the European Union, WHO/FAO, and USDA. The study clearly demonstrates that mycotoxins can be completely detoxified using HPAS at CCC adjusted to pH 4.0 or below. This can be widely applied or integrated into many agricultural and production processes in the food, pharmaceutical, medical, chemical, and nutraceutical industries where pressurized steaming can be applied for the successful detoxification of mycotoxins.

## Graphical abstract

The HPAS process either completely detoxified the mycotoxins or at least reduced them to levels below the maximum limits of 4.00–6.00, 2.00, 2.00, 5.00, and 100 μg/kg for AT, AFB1, AFG1, OTA, and citrinin, respectively, set by the European Union, WHO/FAO, and USDA. The study clearly demonstrates that mycotoxins can be completely detoxified using HPAS at CCC adjusted to pH 4.0 or below. MC = mycotoxin content of the raw (unprocessed) maize samples; M5.0 = mycotoxin content of the maize samples after high‐pressure acidified steaming adjusted to pH 5.0; M4.5 = mycotoxin content of the maize samples after high‐pressure acidified steaming adjusted to pH 4.5; M4.0 = mycotoxin content of the maize samples after high‐pressure acidified steaming adjusted to pH 4.0; GC = mycotoxin content of the raw (unprocessed) groundnut (peanut) samples; G5.0 = mycotoxin content of the groundnut (peanut) samples after high‐pressure acidified steaming adjusted to pH 5.0; G4.5 = mycotoxin content of the groundnut (peanut) samples after high‐pressure acidified steaming adjusted to pH 4.5; and G4.0 = mycotoxin content of the groundnut (peanut) samples after high‐pressure acidified steaming adjusted to pH 4.0.

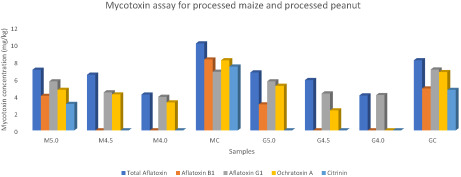

## 1 INTRODUCTION

Mycotoxins are toxic fungal metabolites produced by some molds in grains, nuts, spices, dried fruits, etc. Mycotoxins are chemically stable, with most of them posing concerns to humans and livestock, including the potent toxic mycotoxins such as aflatoxins, ochratoxins, zearalenone, nivalenol, deoxynivalenol, citrinin, fumonisins, and patulin (Gab‐Allah et al., 2023; Qing et al., 2022; Vylkova, 2017). Many industrial and domestic processes, including heat treatment (roasting, boiling, and frying), fermentation, and irradiation, among others, have been studied for the removal of mycotoxins in foods (Awuchi, Ondari, Ofoedu, et al., 2021; Ozkan et al., 2023). However, most of these studies reported incomplete or insufficient elimination of these mycotoxins. Mycotoxins are very stable and can withstand the rigors of food processing, thus requiring a technical approach designed for their removal (Bulgaru et al., 2021). Ensuring food and agro‐safety/quality is very important to not just food and agricultural industries but also to pharmaceutical and biomedical industries (Awuchi, 2023; Saeed et al., 2023).

The study aimed at addressing the increasing problems of mycotoxins worldwide, especially in developing and underdeveloped regions of the world. A widely applicable, cost‐effective, and safe method was considered for the thorough decontamination of mycotoxins from foods, to protect public health and animal safety, and successfully demonstrated that high‐pressure acidified steaming (HPAS) can be a reliable method to solve the problem of mycotoxin exposure from dietary and pharmaceutical sources, which are the major sources of human and animal exposure to mycotoxins and their toxic potency. High‐pressure acidified steaming is a combination of three techniques (high‐pressure [physical], steaming [physical], and acidification [chemical]) with the aim of complete detoxification of the mycotoxins. Citric acid is an edible organic acid used in many products directly consumed by humans, and as such, poses no toxicity when present in normal doses in human foods and animal feed. In this study, we developed a citric acid concentration‐dependent high‐pressure acidified steaming to successfully detoxify mycotoxins, with specific focus on total aflatoxins (AT), aflatoxins B_1_ (AFB_1_), aflatoxin G_1_ (AFG_1_), ochratoxin A (OTA), and citrinin in foods and feeds. The outcome of this study can be widely applied or integrated into many agricultural and production processes in the food, pharmaceutical, medical, chemical, and nutraceutical industries for the successful detoxification of mycotoxins, such as aflatoxins, citrinin, ochratoxins, deoxynivalenol, and fumonisins.

## 2 MATERIALS AND METHODS

### 2.1 Study site and sampling area

Samples were randomly drawn from different farm/market outlets in Kampala. The samples were picked using a stainless‐steel container and taken to the laboratory for further analyses and processing. The samples were selected based on the fresh grain produce from farms that were aimed at supplying and/or selling to the public or industries.

### 2.2 Sample collection and preparation

Three representative samples (three each from three different locations) were randomly collected for each of maize and peanuts from three different agro‐markets in Kampala. The samples were separated into two categories (raw and processed). Processed samples were treated using high‐pressure acidified steaming (HPAS) at different concentrations of citric acid adjusted to pH 4.0, 4.5, and 5.0. Both processed and raw samples were ground into flour using Art's‐Way portable roller mill (PRM30: USA) and labeled accordingly as directed by AOAC (2000a, 2000b). Maximum particle size reduction and thoroughness of mixing of the samples' flour were ensured to achieve effective distribution of contaminated portions.

### 2.3 High‐pressure acidified steaming

The grain flours were subjected to high‐pressure acidified steaming (HPAS) using autoclave, made by Thermo Fisher Scientific, Waltham, MA, United States, at the pressure of 15 PSI at steaming temperature, at various concentrations of citric acid adjusted to pH of 4.0, 4.5, and 5.0. Before steam generation, aqueous citric acid was added to pure water to acidify the water before steam generation and the pH was clearly noted at 4.0, 4.5, and 5.0. The method described by Jin et al. (2017), Jessica (2019), and Maya and Rao (1998) was used with slight modification.

### 2.4 Mycotoxin assay

The ELISA kit method for mycotoxins analysis (AOAC, 2000a, 2000b) was employed to determine the concentration of the respective mycotoxins in the samples. Twenty gram of each sample were grinded and added to 100 mL of 70% methanol blended for 3 min. The solutions were filtered through Whatman No. 1 filter and supernatant was collected. Fifty microliter of the filtrate per well was used for the test. Fifty microliter of each of the respective standards (for each mycotoxin assayed) and test samples were added, respectively, to antibody (mycotoxin)‐coated microtiter plate wells. The plates were sealed, gently homogenized, and incubated for 30 min at 37°C. Three hundred (300 μL) of wash buffer was added to each well and washed three times, and the plates were inverted on a layer of absorbent towels to remove residual water. One hundred microliter of HRP conjugate was added to each antibody‐coated well and incubated at ambient temperature for 30 min. After the incubation period, the plates were washed again with the wash buffer, and the plates were inverted on a layer of absorbent towels to remove residual water. One hundred microliter of substrate reagent was added to each well and then gently mixed thoroughly. This was then incubated at 37°C for 15 min in dark. Subsequently, 100 μL of stop solution was added to each well and gently mixed and the result read within 5 min after addition of stop solution. The optical density (OD) value of each well was determined at 450 nm (reference wavelength 630 nm) using a microplate reader. The values (corresponding to the concentration of the samples) were extrapolated from a standard curve obtained by plotting the absorbance percentage of each standard on the *y*‐axis against the log concentration on the *x*‐axis.

$$\text{Absorbance}\left(\%\right)=A/A_{0}\times 100\%$$

*A*: Average absorbance of standard or samples; *A*_0_: Average absorbance of Standard.

### 2.5 Statistical analysis

The statistical analysis was done using SPSS. Analysis of variance (ANOVA) was conducted to analyze the data and check for any significant differences. Where *p* < .05, the differences were considered to be significant. Where there was significant difference in means, the least square difference (LSD) was done to separate the means. The values are presented as means and standard deviation.

## 3 RESULTS AND DISCUSSION

The results, their interpretation, and discussion are presented in this chapter in detail. The statistical analysis is also explained.

### 3.1 Mycotoxins

The results of the mycotoxins are shown in Table 1. The mycotoxins selected for this study include AT, AFB_1_, AFG_1_, OTA, and citrinin. The removal of these mycotoxins in foods will most likely signify the removal of all the mycotoxins in the foods. The effects of the high‐pressure acidified steaming on the mycotoxins were analyzed and reported in this section.

**TABLE 1:** High‐pressure acidified steaming with citric acid detoxified mycotoxins.

Sample	Total aflatoxin (μg/kg)	AFB_1_ (μg/kg)	AFG_1_ (μg/kg)	Ochratoxin A (μg/kg)	Citrinin (μg/kg)
M5.0	7.02^c^ ± 0.02	4.02^c^ ± 0.00	5.68^c^ ± 0.02	4.72^d^ ± 0.01	3.09^c^ ± 0.01
M4.5	6.44^e^ ± 0.05	ND	4.43^d^ ± 0.02	4.19^e^ ± 0.02	ND
M4.0	4.18^g^ ± 0.02	ND	3.91^g^ ± 0.01	3.26^f^ ± 0.02	ND
MC	10.06^a^ ± 0.02	8.21^a^ ± 0.01	6.79^b^ ± 0.00	8.11^a^ ± 0.02	7.39^a^ ± 0.01
G5.0	6.71^d^ ± 0.01	3.03^d^ ± 0.02	5.68^c^ ± 0.02	5.17^c^ ± 0.01	ND
G4.5	5.82^f^ ± 0.00	ND	4.30^e^ ± 0.14	2.34^g^ ± 0.02	ND
G4.0	4.07^h^ ± 0.02	ND	4.11^f^ ± 0.00	ND	ND
GC	8.11^b^ ± 0.01	4.88^b^ ± 0.01	7.04^a^ ± 0.02	6.75^b^ ± 0.01	4.71^b^ ± 0.00
LSD	0.09	0.41	0.10	0.36	0.84
Limits*	4.00–6.00	2.00	2.00	5.00	100

*Note*: The values are means and standard deviations of the test samples. Mean with different superscripts are significantly different at *p* ≤ .05. MC and GC, mycotoxin content of the raw maize and groundnut (peanut) samples, respectively; M5.0 and G5.0, mycotoxin content of the maize and peanut samples, respectively, after HPAS at CCC adjusted to pH 5.0; M4.5 and G4.5, mycotoxin content of the maize and peanut samples, respectively, after HPAS at CCC adjusted to pH 4.5; M4.0 and G4.0, mycotoxin content of the maize and peanut samples, respectively, after HPAS at CCC adjusted to pH 4.0. LSD, the least significant difference. ND, not detected, that is, no mycotoxin was found.

* The limits are the maximum allowable limits (μg/kg in foods such as grains and nuts) in the European Union, WHO/FAO, and USDA (Agriopoulou et al., 2020; Awuchi, Ondari, Ogbonna, et al., 2021; EFSA, 2020; European Commission, 2019; Giovati et al., 2015).

### 3.2 Aflatoxins

The AT, AFB_1_, and AFG_1_ before and after processing of the samples were determined and the results are shown Table 1. The mean values of the AT, AFB_1_, and AFG_1_ in the raw samples were 10.06 ± 0.02, 8.21 ± 0.01, and 6.79 ± 0.00 μg/kg for maize, respectively (*p* ≤ .05); for groundnut (peanut), they were 8.11 ± 0.01, 4.88 ± 0.01, and 7.04 ± 0.02 μg/kg, respectively. These values in this study are far above the maximum limits of 4.00, 2.00, and 2.00 μg/kg for AT, AFB_1_, and AFG_1_, respectively, in grains and grain products based on the maximum limits set by the European Union, WHO/FAO, and USDA (Agriopoulou et al., 2020; Awuchi, Ondari, Ogbonna, et al., 2021; EFSA, 2020; European Commission, 2019; Giovati et al., 2015). After processing at different citric acid concentrations, adjusted to pH gradients of 4.0, 4.5, and 5.0, the values of the mycotoxins significantly reduced and were not detected in some samples (*p* ≤ .05). At citric acid concentration adjusted to pH 5.0, the AT, AFB_1_, and AFG_1_ in the samples significantly reduced to 7.02 ± 0.02 (30% reduction), 4.02 ± 0.00 (51%), and 5.68 ± 0.02 (16%) μg/kg, and 6.71 ± 0.01 (17%), 3.03 ± 0.02 (38%), and 5.68 ± 0.02 (19%) μg/kg for maize and groundnut, respectively. At citric acid concentration adjusted to pH 4.5, AFB_1_ was not detected in both samples (i.e., 100% detoxification of AFB_1_ was achieved), while the AT and AFG_1_ in the samples significantly reduced to 6.44 ± 0.05 (36%) and 4.43 ± 0.02 (35%) μg/kg, and 5.82 ± 0.00 (28%) and 4.30 ± 0.14 (39%) μg/kg for maize and groundnut, respectively. Similarly, when the citric acid concentration was increased and adjusted to pH of 4.0, AFB_1_ was also not detected in both samples (i.e., same 100% detoxification of AFB_1_ was achieved), while AT and AFG_1_ significantly reduced to 4.18 ± 0.02 (59%) and 3.91 ± 0.01 (42%) μg/kg, and 4.07 ± 0.02 (50%) and 4.11 ± 0.00 (42%) μg/kg for maize and groundnut, respectively. After HPAS processing at citric acid concentration adjusted to pH 4.0, the mycotoxins either completely detoxified or reduced to safe levels, except for AFG_1_ which was relatively higher than the maximum allowable limit. These levels can be further reduced or completely detoxified by increasing the citric acid concentrations in the high‐pressure steam and adjusting to pH below 4.0 (*p* ≤ .05). This study has shown reliable model for completely detoxifying aflatoxins or at least reducing them to safe levels. This shows the effectiveness of high‐pressure acidified steaming (HPAS) is completely detoxifying mycotoxins or at least significantly reducing them to safe levels; the efficiency of the detoxification by HPAS is citric acid concentration dependent. The values before processing are comparable to the values reported in other studies. Kholif et al. (2021) reported average contamination levels of 26.9 μg/kg and 2.65 μg/kg in total for AFB1, AFB2, AFB_1_, and AFG_1_ in sunflower oilseed and corn samples, respectively. Qing et al. (2022) reported a value of 20.10 mg/kg AFB_1_ in feed intake in their study. The differences in these values compared to the values in my raw samples may be due to the methods used by Kholif et al. (2021), who made use of size exclusion chromatography followed by HPLC. AFB_1_ is the most known toxic of all mycotoxins; its removal in foods is very important to food safety and animal/human health. The mean values of the total aflatoxins reported for maize in this study are slightly higher than the mean value of 3.14 ± 3.01 μg/kg reported by Awuchi et al. (2020), but the mean values for peanut in this study are lower than the mean value of 26.30 ± 11.47 μg/kg reported in the same study. These differences may be due to differences in the locations of the sample collection.

In previous studies, aflatoxins, including AFB_1_ and AFG_1_, have been shown to be nephrotoxic, immunotoxic, teratogenic, carcinogenic, mutagenic, hepatotoxic, neurotoxic, and genotoxic, among various toxic potencies. This study provides interesting knowledge into how these toxic effects can be zeroed or significantly reduced by applying HPAS against these mycotoxins in foods, especially grains. El‐Mahalaway (2015) and Joubrane et al. (2020) described how AFB_1_ exposure in adult male albino rats' renal cortex resulted in necrotic changes and degeneration with disrupted basal lamina, glomerular atrophy with light elimination from their capillaries, and enlargement with glomeruli luminal dilation. Li et al. (2018) and Wu et al. (2021) reported the testing of aflatoxins in many renal cell lines for a better understanding of their toxic mechanisms, with “primary fetal bovine kidney cell” and the “MadineDarby bovine kidney cell.” Aflatoxins exerted toxic potencies via several mechanisms. Several recent studies have described the various mechanisms involved in aflatoxins toxicities (Awuchi, Nwozo, et al., 2022; Lai et al., 2022) and their deleterious toxic potency (An et al., 2017; Awuchi, Ondari, et al., 2022). These toxic potencies can be avoided by treating grains with HPAS at the appropriate CCC before consumption. AFB_1_ potentiates autophagy mediated by ROS in RAW264.7 and THP‐1 cell lines (An et al., 2017; Lai et al., 2022). Navale et al. (2021) described the studies done on AFB_1_, which mostly focused on evaluating its teratogenic effects on chicks, rats, chickens, and eggs, and found teratogenic activities. Mohamed et al. (2022) reported that ozone can be used as a solution to eliminate aflatoxins' risk in meat and meat products (kofta and luncheon). In their study, they reported that raw Kofta and luncheon samples contained 15.2 ppb and 4.8 ppb of total aflatoxins, respectively (Mohamed et al., 2022), which is significantly different compared to the mean values of 10.06 ± 0.02 and 8.11 ± 0.01 reported in this study for raw maize and peanut samples, respectively. Mohamed et al. (2022) reported that the degree of detoxification depends on the concentration of ozone exposed to the samples. This concentration‐dependent detoxification is similar to the citric acid concentration‐dependent detoxification reported in this study. In Mohamed et al. (2022) study, at 20 ppm ozone concentration, the most detoxified aflatoxins were AFG_1_ (68.3%) and AFB_2_ (67.1%), while other aflatoxins reduced in ranges of 61.4% (44.7 ppb) and 55.2% (11.6 ppb) for kofta and luncheon, respectively; at 40 ppm ozone concentration, the most detoxified aflatoxins were AFB_2_ (91.7%) and AFG_1_ (100%), while other aflatoxins reduced by ranges from 85.7% (54.6 ppb) and 78.4% (61.4 ppb), respectively. Simões et al. (2023) reported Brazilian table olives as a source of lactic acid bacteria with antifungal and antimycotoxigenic activity. The methods used in this study can complement this treatment in an effort to completely detoxify these mycotoxins.

Due to the persistence of mycotoxins such as AFB_1_ and AFG_1_, in foods and feeds, many other studies have been done recently with the aim of harnessing novel ways to control these biological toxins (Awuchi, Ondari, Ofoedu, et al., 2021; Chinaza et al., 2021). The effectiveness of the method developed in this study demonstrates comparative advantage, including cost‐effectiveness, in removing mycotoxins compared with many methods that have been used in previous studies. This method also augments other methods that have been studied, including the promising ones that may not be applied to all foods. Adebo et al. (2019) studied the effect of fermentation on mycotoxins in sorghum and ting and reported promising results. Another recent study was done by Lorán et al. (2022), aimed at evaluating the in vitro effect of essential oils from *Origanum virens, Rosmarinus officinalis*, and lavandins *Grosso* and *Abrial*, as well as natural phenolic acids, such as chlorogenic, caffeic, p‐coumaric, and ferulic acids, on the production of AFB_1_, AFB_2_, AFG_1_, and AFG_2_. Lorán et al. (2022) reported a significant reduction in levels of aflatoxins after treatment with essential oils and phenolic acids, although some did not have any effect. The initial values of AFB_1_, AFB_2_, AFG_1_, and AFG_2_ in their raw samples were 0.52, 0.09, 0.94, and 0.26 μg/mL, respectively. These values are less than the values found in the raw samples in this study. Park (2002) reported a 40%–80% reduction in aflatoxins achieved using physical cleaning by removing damaged, mold‐infested nuts, seeds, or kernels from the whole grains. Although the levels of aflatoxins still remained unsafe after this removal. In addition to the physical cleaning, the method developed in this study can be used to either completely remove these mycotoxins or at least reduce them to safe levels. Kaushik (2015) evaluated the effects of food processing operations on my mycotoxin detoxification, including extrusion, roasting, flaking, frying, baking, cleaning, cooking, sorting, and trimming, and reported that processing operations such as thermal, physical, and chemical conditions play important role in detoxifying mycotoxins, with high‐temperature processes having more effects. However, they also concluded that all the processes evaluated significantly reduced the concentrations of mycotoxins, but did not completely eliminate them (Kaushik, 2015). This gives the HPAS method used in this study a comparative advantage, as it can completely detoxify most mycotoxins at CCC adjusted to pH 4 or below.

### 3.3 Ochratoxin A

The values of OTA in the raw samples were 8.11 ± 0.02 and 6.75 ± 0.01 μg/kg for maize and groundnut (peanut), respectively (see Table 1). These values are far higher than the maximum limits of 5 μg/kg for OTA in grains and grain products set by the European Union, WHO/FAO, and USDA (Agriopoulou et al., 2020; EFSA, 2020; Giovati et al., 2015). After HPAS processing at citric acid concentration adjusted to pH 5.0, the OTA levels significantly decreased to 4.72 ± 0.01 μg/kg (42% detoxification) and 5.17 ± 0.01 μg/kg (23% detoxification) for maize and groundnut, respectively (*p* ≤ .05). At citric acid concentration adjusted to pH 4.5, the OTA further significantly decreased to 4.19 ± 0.02 μg/kg (48% detoxification) and 2.34 ± 0.02 μg/kg (65% detoxification) for maize and groundnut, respectively. Interestingly, after the citric acid concentration was adjusted to pH 4.0, OTA was not detected in groundnut (100% elimination/detoxification), and in maize, it significantly decreased to 3.26 ± 0.02 μg/kg (60% detoxification) (*p* ≤ .05). The high‐pressure acidified steaming process either completely detoxified the OTA or at least reduced it to levels far below the maximum limit of 5 μg/kg for OTA set by the European Union, WHO/FAO, and USDA. It was observed that increasing the citric acid concentration significantly reduced the levels of OTA in the grain samples in a manner that shows that increasing the citric acid concentration in pressured steaming process can completely detoxify mycotoxins such as OTA in foods and feeds; that is, the efficiency of the detoxification by HPAS is citric acid concentration dependent (*p* ≤ .05). The values in the raw samples are relatively lower than the average value of 101.41 mg/kg of OTA in samples from feed intake reported by Qing et al. (2022). OTA and aflatoxins often cooccur in foods and feeds, often along with other mycotoxins (Agriopoulou et al., 2020; Awuchi et al., 2020). Their removal is very important for food safety and public health. This study has developed a reliable method and processing regimen for completely detoxifying mycotoxins in foods and feeds or at least reducing them to safe levels (i.e., levels at which the body can easily detoxify them without them exerting any form of toxicity). Awuchi et al. (2020) and Awuchi, Owuamanam, and Ogueke (2021) reported the effects of ochratoxins on the nutritional and functional properties of foods. Gan et al. (2017) reported that in vitro OTA nephrotoxic effects in primary porcine splenocytes and PK15 cells showed that 0.5–4.0 and 2.0–8.0 mg/mL per day, respectively, induced apoptosis and cytotoxicity by phosphorylation and signaling of ERK and p38. These toxic effects can be avoided by employing the HPAS method to completely detoxify OTA or reduce its levels to safe levels.

OTA exerts many toxic effects in humans and animals, including nephrotoxic, hepatotoxic, carcinogenic, immunotoxic, neurotoxic, genotoxic, and teratogenic effects, among other toxic effects. Loboda et al. (2017) reported the nephrotoxic effects of OTA on porcine tubular epithelial cells after Nrf2 inhibition with reduced vascular endothelial growth factor and claudin‐2 levels and increased expression of proapoptotic, profibrotic, and proinflammatory factors. In other studies, DNA microarray analysis following the proximal tubular cells double fluorescence labeling of primary rat and Wistar rats treated with several ochratoxin A doses showed transcriptional changes in genes involved in apoptosis, inflammatory reactions, and DNA damage responses (Gan et al., 2022; Ráduly et al., 2021). Real‐time polymerase chain reactions application in studying the expression of gene responsible for cell division and cell control in OTA‐treated male F344 rats at doses of 210, 70, 21, and 0 mg/kg of body weight showed that OTA induces the expression of excess mitosis key regulators, such as aurora B kinase, cyclin's kinase‐dependent inhibitors, serine/threonine protein kinase PLK1, surviving, topoisomerase II, cyclin‐dependent kinase 1 (Cdk1Cdc2), and cyclins with some key regulators' upregulation in the proximal tubular S3 cell, where OTA‐induced tumors appear (EFSA Panel on Contaminants in the Food Chain (CONTAM) et al., 2020; Khoi et al., 2021). High and medial doses of ochratoxin A exert liver toxicity. In a study involving OTA toxicity on HepG2 cell lines, Gayathri et al. (2015) reported increase in intracellular ROS accompanied by breaks in DNA strands and mitochondria‐mediated intrinsic apoptosis. Neural stem/progenitor cells (NSCs) formulated from adult mouse brains' hippocampus were tested for their OTA vulnerability in vitro, and 0.01–100 mg OTA/mL concentrations showed that OTA causes time‐ and dose‐dependent (6–72 h) reduction in differential and proliferative viability; differentiated neurons have less vulnerability to toxins compared to proliferating NSCs (Bhat et al., 2016; Gill & Kumara, 2019). These toxicities can simply be avoided by either eliminating or at least reducing the levels of OTA using high‐pressure acidified steaming methods as described in this study.

Many methods have also been explored in other studies in an attempt to reduce the toxic effects of ochratoxin A and other mycotoxins. Leitão and Enguita (2021) conducted research on filamentous fungal proteomes' enzymes that can degrade ochratoxins in a systematic structure‐based manner. They concluded that filamentous fungi are rich in hydrolases that can potentially degrade ochratoxins, and may detoxify many food commodities. However, the successful application of these enzymes in real‐time is yet to be ascertained. The method developed in this study can easily be applied in real time and large‐scale food and agricultural production, as many industries already make use of steam generation and application in many processes.

### 3.4 Citrinin

Table 1 shows that the values of citrinin in the raw samples were 7.39 ± 0.01 and 4.71 ± 0.00 μg/kg for maize and groundnut (peanut), respectively. There were significant differences in the levels of citrinin in the raw samples, as shown in Table 1. It was observed that these values are within the maximum limit of 100 μg/kg set by the European Union and WHO/FAO (EFSA, 2020; EFSA Panel on Contaminants in the Food Chain (CONTAM) et al., 2020). After HPAS processing at citric acid concentration adjusted to pH 5.0, the level of citrinin in maize significantly reduced to 3.09 ± 0.01 μg/kg (58% detoxification), while it was not detected in groundnut (100% elimination/detoxification). Very interestingly, at HPAS processing of citric acid concentration adjusted to pH 4.5 and 4.0, no citrinin was detected thereafter (100% elimination/detoxification). This shows that the HPAS processing method completely eliminates/detoxifies mycotoxins such as citrinin in foods and feeds. The mean values reported in the raw samples are relatively less than the mean values of 14.6–23.8 μg/kg in corn and wheat samples reported by Čulig et al. (2017), who analyzed the citrinin levels in grains along with its health effects. Many foods, feeds, and supplements have been proven to contain high levels of citrinin. Ali et al. (2015) found that the average urine level for citrinin and its metabolite (dihydrocitrinone) were 0.03 ng/mL and 0.06 ng/mL respectively, which when adjusted to the creatinine level, 20.2 and 60.9 ng/g creatinine for citrinin and dihydrocitrinone, respectively; it became clear that the metabolite appearance in urine was 3× higher. Silva et al. (2020) described various foods that contain citrinin, from low to extremely high amounts, including grains. The presence of citrinin and other mycotoxins can be eliminated or at least significantly reduced to safe levels using the HPAS developed in this study. Magro et al. (2016) simulated citrinin contamination of 625 μg/L and studied its removal using naked magnetic nanoparticles. The results showed that the efficiency of the decontamination using naked magnetic nanoparticles is 70% (Magro et al., 2016), which still leaves citrinin at unsafe levels; this unsafe level can be eliminated or reduced to safe levels using the methods developed in this study. Piemontese et al. (2018) have also described the use of nanoparticles and their magnetic nanocomposites to reduce the levels of mycotoxins in grains. Citrinin is among the rarely studied mycotoxins (Narváez et al., 2021). Tangni et al. (2021) studied citrinin in food supplements produced from red yeast rice and *Ginkgo biloba* leaves and evaluated the citrinin stability under storage. They reported that citrinin values changed after storage at different temperature ranges between 4 and 24°C. Bartkiene et al. (2021) proceed wheat bran by the combination of extrusion (at screw speeds of 25, 20, and 16 rpm, and temperature of 115–130°C) and fermentation using *L. uvarum* and *Lactobacillus plantarum* strains; they reported 29.8 μg/kg total mycotoxin concentration after the combined processing. The HPAS in this study proved more effective in mycotoxin detoxification than many other methods, such as the combined extrusion and fermentation methods.

Citrinin exerts many toxicities on both humans and animals, including genotoxicity, nephrotoxicity, carcinogenicity, immune suppression, acute toxicity, etc. The toxicity of citrinin is further worsened by the fact that it usually occurs along with other mycotoxins, especially OTA and AFB_1_, as they are produced by the same species of fungi. It mostly occurs with OTA, with both synergistically exerting nephrotoxic effects, and may have influence on necrosis and apoptosis in hepatocytes (Gayathri et al., 2015; Jaus et al., 2022). Many in vitro studies showed that citrinin toxicity involves decreased cytokine production nitride oxide gene expression's inhibition, increase in ROS, inhibition of DNA and RNA synthesis, oxidative stress induction, and apoptotic cell death activation through the caspase cascade system and signal transduction pathways (European Food Safety Authority, 2012). Citrinin and its metabolite (dihydrocitrinon) have been detected in urine by Ali et al. (2015) in 82% and 84% of urine samples, respectively. Citrinin mostly targets the kidney (Jaus et al., 2022). These toxic effects of citrinin and other mycotoxins can be prevented by treating foods and feeds using HPAS in citric acid concentration adjusted to pH 4.5 or 4.0 or below prior to further processing and/or consumption.

## 4 CONCLUSION

Mycotoxins are very toxic and persistent in foods and feeds. They escape the rigors of many processes used in food, pharmaceutical, and nutraceutical industries. We developed widely applicable, cost‐effective, and safe method for mycotoxins decontamination, and successfully demonstrated that high‐pressure acidified steaming (HPAS) can be a reliable method to solve the problem of mycotoxin exposure from dietary and pharmaceutical sources. The results of this study showed that the HPAS can completely detoxify mycotoxins, such as AT, AFB_1_, AFG_1_, OTA, and citrinin, or at least reduce them to safe levels. The study strongly recommends and encourages the application of the HPAS method at industrial scale in the food, pharmaceutical, nutraceutical, medical, and chemical industries where citric acid‐containing pressurized steaming can be applied for the detoxification of mycotoxins.

## CONFLICT OF INTEREST STATEMENT

All authors declare no conflict of interest.

## ETHICAL APPROVAL

The study does not involve any human or animal testing.

## Data Availability

Additional data for this study can be made available from the corresponding author upon request.
